# Matrix metalloproteinase 9 and cellular fibronectin plasma concentrations are predictors of the composite endpoint of length of stay and death in the intensive care unit after severe traumatic brain injury

**DOI:** 10.1186/1757-7241-20-83

**Published:** 2012-12-18

**Authors:** Jean-Christophe Copin, Marie My Lien Rebetez, Natacha Turck, Xavier Robin, Jean-Charles Sanchez, Karl Schaller, Yvan Gasche, Bernhard Walder

**Affiliations:** 1 Geneva Neuroscience Center, University of Geneva, Geneva, Switzerland; 2 Division of Intensive Care, University Hospitals of Geneva, Geneva, Switzerland; 3 Division of Neurosurgery, University Hospitals of Geneva, Geneva, Switzerland; 4 Division of Anaesthesiology, University Hospitals of Geneva, Geneva, Switzerland; 5 Biomedical Proteomics Research Group, Department of Human Protein Sciences, University of Geneva Medical Center, Geneva, Switzerland; 6 Centre Médical Universitaire, 1, rue Michel Servet, Genève 4, CH-1211, Switzerland

**Keywords:** Head injury, Prediction, Outcome, Plasmatic biomarker

## Abstract

**Background:**

The relationship between severe traumatic brain injury (TBI) and blood levels of matrix metalloproteinase-9 (MMP-9) or cellular fibronectin (c-Fn) has never been reported. In this study, we aimed to assess whether plasma concentrations of MMP-9 and c-Fn could have predictive values for the composite endpoint of intensive care unit (ICU) length of stay (LOS) of survivors and mortality after severe TBI. Secondary outcomes were the state of consciousness measured with the Glasgow Coma Scale (GCS) of survivors at 14 days and Glasgow Outcome Scale Extended (GOSE) at 3 months.

**Methods:**

Forty-nine patients with abbreviated injury scores of the head region ≥ 4 were included. Blood was sampled at 6, 12, 24 and 48 hours after injury. MMP-9 and c-Fn concentrations were measured by ELISA. The values of MMP-9 and c-Fn, and, for comparison, the value of the GCS on the field of the accident (fGCS), as predictors of the composite outcome of ICU LOS and death were assessed by logistic regression.

**Results:**

There was a linear relationship between maximal MMP-9 concentration, measured during the 6-12-hour period, and maximal c-Fn concentration, measured during the 24-48-hour period. The risk of staying longer than 9 days in the ICU or of dying was increased in patients with a maximal early MMP-9 concentration ≥ 21.6 ng/ml (OR = 5.0; 95% CI: 1.3 to 18.6; p = 0.02) or with a maximal late c-Fn concentration ≥ 7.7 μg/ml (OR = 5.4; 95% CI: 1.4 to 20.8; p = 0.01). A similar risk association was observed with fGCS ≤8 (OR, 4.4; 95% CI, 1.2-15.8; p = 0.02). No relationship was observed between MMP-9, c-Fn concentrations or fGCS and the GCS at 14 days of survivors and GOSE at 3 months.

**Conclusions:**

Plasma MMP-9 and c-Fn concentrations in the first 48 hours after injury are predictive for the composite endpoint of ICU LOS and death after severe TBI but not for consciousness at 14 days and outcome at 3 months.

## Background

The personal and societal consequences of severe traumatic brain injury (TBI) are considerable related to its high rate of occurrence of cognitive dysfunction and disability [1,2]. Its estimated incidence in Europe is between 9–17 per 100'000 person/year [3,4]. The majority of patients are men with a mean age of 40 to 50 years, injured by falls or road traffic accidents [5,6]. The outcome after severe TBI is often unfavorable with one third to one half of patients dying during the 6 following months and one third presenting severe disabilities [6-8]. In recent years validated models were developed to predict poor outcome after TBI [9]. However, predictive power when using such clinical models for individual patients is still limited. All these models use components of the Glasgow Coma Scale (GCS) which is known to have a large inter-individual assessment range [10] and which may explain this limited prediction capacity for individual patients. One research direction in patients with TBI is the attempt to improve these clinical prediction models and to improve overall management via new diagnostic assessments. Neuronal, glial and inflammatory biomarkers were tested to add supplementary information on diagnosis and to provide early information about prognosis on functional and cognitive outcome [11-18]. Among all these biomarkers, S100b, a calcium binding protein with high expression in astrocytes, has been the most extensively investigated. S100b levels predicted mortality or poor outcome [11,19].

Matrix metalloproteinases (MMP) are a family of zinc-dependent endopeptidases involved in a number of neurological diseases, in which neuroinflammation plays a significant role [20]. MMP-9 degrades components of the basal lamina, such as fibronectin and collagen-IV, leading to disruption of the blood–brain barrier and vasogenic brain edema [21-23]. Elevated brain MMP-9 concentration was found in experimental TBI [24-26] and brain lesion volumes were significantly reduced in TBI animals after overexpression of tissue inhibitor of metallaloproteinases-1 or genetic knocked-out of the mmp-9 gene [27,28]. Recently, it was described an acute elevation of MMP-9 concentration in the cerebrospinal fluid of six severe TBI patients as compared to normal pressure hydrocephalus patients, but no differences in plasma concentration between these two groups of patients were observed [29]. Two other studies, carried out on a limited number of patients, showed elevated MMP-9 concentration in the plasma [30,31] and in the brain extracellular fluid [31] during the acute stage of TBI.

Fibronectins are adhesive glycoproteins that promote cell–cell and cell–matrix interactions [32]. Two forms of fibronectins exist: plasma fibronectin, a dimeric and soluble form secreted by hepatocytes directly into the circulation, and cellular fibronectin (c-Fn), a multimeric form assembled into fibrils and found in the extracellular matrix [33]. Because c-Fn is incorporated between endothelial cells and pericytes [34], high plasma levels of this molecule in TBI patients might be indicative of the loss of blood brain barrier integrity. Elevated concentrations of c-Fn were detected in the plasma after major abdominal, thoracic or pulmonary traumatic injury [35]. However, to our knowledge, no study has reported the release of c-Fn into the circulation after TBI.

Blood levels of MMP-9 and c-Fn have good predictive values for the evaluation of hemorrhagic transformation after thrombolytic therapy in acute ischemic stroke [36]. However, the relationship between TBI outcome and blood levels of MMP-9 and c-Fn has never been reported. In this study, we investigated the hypothesis that plasma MMP-9 and c-Fn concentrations could play also a role in TBI patients in the early period after injury, and, in particular, could have a predictive value for certain outcomes such as intensive care unit (ICU) length of stay (LOS) and mortality after severe TBI.

## Methods

### Patients

We analysed the data of consecutive patients enrolled in a prospective study on “Patient-relevant Endpoints after Brain Injury from Traumatic Accidents” between May 2007 and November 2009. The study was approved by the ethical committee of the Geneva University Hospital, Geneva, Switzerland and was a follow up investigation of our previous study [8]. Written informed consent by proxy within 14 days after injury replaced informed consent by patients, who were all severely injured at the time of enrolment.

Patients with TBI from blunt trauma were enrolled and included if severe TBI was confirmed by cerebral CT scan. Severe TBI was defined by the presence of an abbreviated injury scores (AIS) of the head region (HAIS) ≥ 4 within the first 24 hours (first cerebral CT or following CT within 24 hours after injury). For AIS assessment, we used the 1990 revision, update 1998 [37]. On the 6-point scale of AIS, values of 4 to 6 correspond to severe to fatal lesions.

### Outcomes

The primary outcome was the composite endpoint ICU LOS for survivors and mortality in the ICU.

Secondary outcomes included ICU LOS for survivors, mortality before 3 months, the state of consciousness (Glasgow Coma Scale) at 14 days (GCS > 8 = conscious) and Glasgow Outcome Scale Extended (GOSE) at 3 months [38]. Contrary to the hospital LOS where readiness of discharge and real discharge may be different, patients always left the ICU without delay; i.e. when their medical conditions permitted them to be discharged. Therefore, this ICU LOS as endpoint was investigated.

### Predictive factors

The relationships between the potential predictive factors MMP-9, c-Fn and the established predictive factor GCS on the field of the accident (fGCS), as reported by out of hospital emergency medical services were assessed [39].

In addition, other early clinical and radiological parameters such as cerebral edema, midline shift, subarachnoid hemorrhage (SAH), intraventricular hemorrhage (IVH) or abnormal pupil reactions on the field of the accident were assessed.

### MMP-9 and c-Fn enzyme-linked immunosorbent assays

Blood was sampled at 6, 12, 24 and 48 hours after injury in EDTA tubes and centrifuged. Plasma was stored at −80°C until analysis. Plasma MMP-9 and c-Fn concentrations were measured blindly by enzyme-linked immunosorbent assays (biotrack Elisa system, Amersham #RPN2614; Elisa kit for human c-Fn, Biohit Oyj #603010) according to the manufacturer’s instructions.

### Statistical analysis

Data were expressed as medians (interquartile ranges). Boxplots show median values, lower and upper quartiles, and the largest and smallest observed values (whiskers) if the values lay within 2.5 time the interquartile range. Multiple group comparisons of related samples were done using the Friedman's 2-Way ANOVA by Ranks test followed by Wilcoxon Matched-Pair Signed-Rank tests. Independent samples were compared using the Mann–Whitney U test for 2 samples. Linear regression was used to assess the relationship between MMP-9 and c-Fn concentrations. Receiver operating characteristic (ROC) curves and logistic regression were used to assess the values of MMP-9, c-Fn and fGCS as predictors of prolonged ICU stay or death and to determine odds ratios (OR) and sensitivity/specificity. Statistical analyses were performed with PASW Statistics release 18 for the Macintosh. P < 0.05 was considered significant. ROC curve analyses were performed with the pROC package for TIBCO Spotfire S+ version 8.2 for Windows. Comparison between paired ROC curves were computed with the bootstrap test and 10 000 replicates as described previously [40].

## Results

### Clinical characteristics of the cohort and outcome

Forty-nine patients were included (13 patients (27%) with a HAIS of 4 and 36 patients (73%) with a HAIS of 5). The median fGCS was 7 (3–10) and 40.0% of the patients had abnormal pupil reaction (Table 1). The median age of the cohort was 39 years (26–55). Six patients (12%) had multiple traumas with an AIS of the chest region of 4 (5 patients: 4 died, 1 survived) or an AIS of the abdomen region of 4 (1 patient who survived).

**Table 1:** Clinical characteristics of the cohort

**Characteristics of the cohort**	**Values**	**n**
Age (years)	39 (25–55)	49
Height (m)	1.75 (1.70–1.80)	29
Weight (kg)	70 (66–80)	31
GCS on scene	7.0 (3.0–10.0)	49
Abnormal pupil reaction on scene	18/45 (40.0%)	45
Alcohol smell	15/46 (32.6%)	46
Intubation on scene	38/49 (77.6%)	49
GCS in emergency department	3.0 (3.0 – 3.0)	46
Abnormal pupil reaction in emergency department	18/46 (39.1%)	46
SAPS II	52.0 (44.5–56.5)	39
ISS	29 (25–34)	49
Mortality at 14 days	11/49 (22.4%)	49
GCS at 14 days	11.0 (3.0–15.0)	49
GCS of survivors at 14 days	13.5 (8.0–15.0)	38
Abnormal pupil reaction of survivors at 14 days	4/34 (11.8%)	34
Mortality at 3 months	15/49 (30.6%)	49
GOSE at 3 months	3.0 (1.0–4.0)	37
GOSE of survivors at 3 months	3.5 (3.0–4.0)	22
LOS of survivors in ICU (days)	9.0 (4.0–15.0)	37
LOS of survivors in acute hospital (days)	15.5 (10.0–24.5)	36

The median ICU LOS of the survivors was 9.0 days (4.0-15.0); median hospital LOS was 15.5 days (10.0–24.5). Fifteen patients (30.6%) died before 3 months. The median GCS of the survivors at 14 days was 13.5 (8–15). The median GOSE of the survivors at 3 month was 3.5 (3–4).

### Temporal profiles of plasma MMP-9 and c-Fn concentrations

Six hours after TBI, plasma MMP-9 concentration was 25.9 ng/ml (9.8–60.1) (Table 2). MMP-9 concentration decreased significantly over the next days, to reach a final value of 6.4 ng/ml (1.4–19.3) 48 hours after injury (*p* = 0.0008).

**Table 2:** Plasma MMP-9 and c-Fn concentrations

**Time after TBI (h)**	**MMP-9 (ng/ml)**	P *	**c-Fn (μg/ml)**	P *	**n**
6	25.9 (9.8–60.1)		3.3 (2.4–6.1)		26
12	16.1 (7.8–31.2)	0.01	4.4 (2.6–6.3)	0.17	40
24	9.3 (5.2–20.0)	0.02	5.3 (2.7–8.5)	0.0001	45
48	6.4 (1.4–19.3)	0.0008	5.3 (3.5–9.3)	0.005	35

* As compared to the 6-hour time point.

Six hours after TBI, plasma c-Fn concentration was 3.3 μg/ml (2.4-6.1) (Table 2). c-Fn concentration increased significantly over the next days, to reach a final value of 5.3 μg/ml (3.5-9.3) 48 hours after injury (*p* = 0.005).

**Figure 1:**
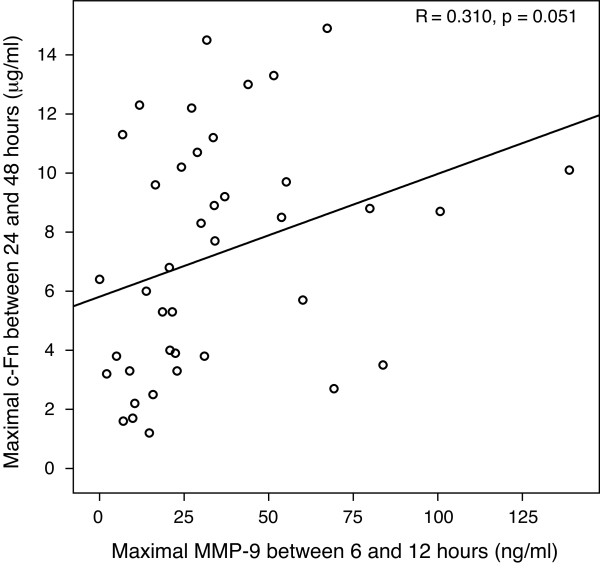
**Relationship between early MMP-9 concentration and subsequent c-Fn concentration.** Maximal MMP-9 concentration measured between 6 and 12 hours was plotted against maximal c-Fn concentration measured between 24 and 48 hours.

**Figure 2:**
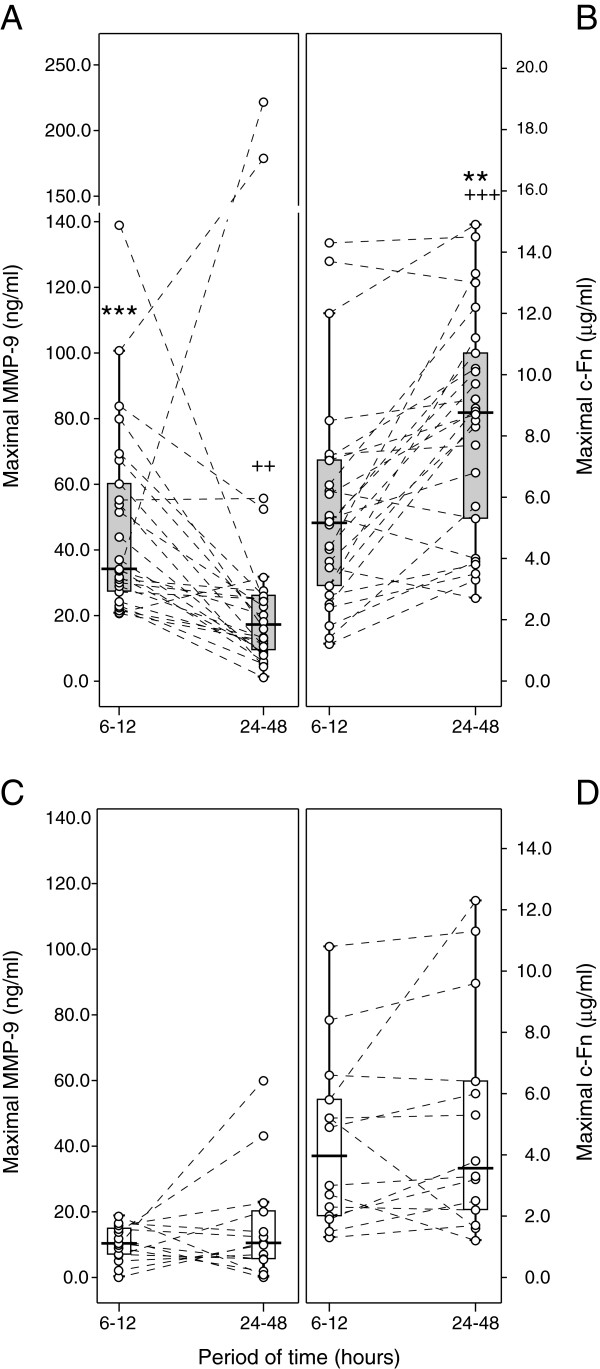
**Variations of MMP-9 and c-Fn concentrations during the 48 hours following TBI.** Patients were grouped according to the maximal MMP-9 concentration between 6 and 12 hours. Grey boxplots: group of patients with maximal early MMP-9 concentration ≥ 20 ng/ml, white boxplots: group of patients with maximal early MMP-9 concentration < 20 ng/ml. **A** and **C**: maximal MMP-9 concentration between 6 and 12 hours , **B** and **D**: maximal c-Fn concentration between 24 and 48 hours. Each line represents a single patient. ** *p* < 0.01, *** *p* < 0.001 as compared to the opposite group of patients at the same period. ++ *p* < 0.01, +++ *p* < 0.001 as compared to the 6–12 hour period within the same group.

There was a linear relationship between maximal MMP-9 concentration measured between 6 and 12 hours and maximal c-Fn concentration measured between 24 and 48 hours (Figure 1). The higher the MMP-9 concentration between 6 and 12 hours, the higher the subsequent c-Fn concentration between 24 and 48 hours (r = 0.310, *n* = 40, *p* = 0.05). Moreover, by grouping patients according to maximal MMP-9 concentration between 6 and 12 hours, we observed a c-Fn concentration increase over the first 48 hours only in patients with an early MMP-9 concentration ≥ 20 ng/ml (Figure 2).

### Relationship between the potential predictive factors MMP-9 and c-Fn and the established predictive factor f GCS

A trend toward higher maximal early MMP-9 concentration (+42%, *p* = 0.13, *n* = 43) or maximal late c-Fn concentration (+83%, *p* = 0.10, *n* = 46) was observed in patients with a fGCS ≤ 8 as compared to patients with a fGCS > 8 (Table 3).

**Table 3:** Relationship between MMP-9, c-Fn or fGCS and different clinical parameters

**Parameters**		**Maximal MMP-9 between 6–12 hours**			**Maximal c-Fn between 24–48 hours**			**fGCS**		
		ng/ml	*P*	*n*	μg/ml	*P*	*n*	Score	*P*	*n*
Oedema	No	22.0 (11.8–51.5)	0.24	22	6.0 (3.5–8.9)	0.24	25	8.5 (4.0–12.0)	0.007	26
	Yes	31.0 (20.6–43.9)		21	8.3 (3.9–11.2)		21	4.0 (3.0–7.5)		23
Midline shift (mm)	< 5	24.2 (15.8–55.2)	0.52	29	6.6 (3.8–10.1)	0.64	30	7.0 (4.0–10.0)	0.28	33
	≥ 5	25.9 (13.8–34.1)		14	6.9 (3.6–10.2)		16	4.0 (3.0–8.5)		16
SAH	No	20.8 (9.8–34.1)	0.21	13	4.7 (3.0–9.2)	0.26	12	10.0 (6.0–12.0)	0.03	13
	Yes	29.5 (16.3–53.8)		30	7.2 (3.9–10.2)		34	5.0 (3.0–8.5)		36
IVH	No	24.2 (13.8–53.8)	0.71	33	7.6 (3.7–10.0)	0.59	35	7.0 (3.0–10.0)	0.99	37
	Yes	25.9 (20.8–34.1)		10	4.0 (3.9–8.9)		11	5.5 (3.0–11.0)		12
fGCS	9-15	21.5 (11.8–28.9)	0.13	13	4.6 (3.2–7.7)	0.10	14	11.5 (10.0–14.5)	0.000	16
	3-8	30.5 (16.5–53.8)		30	8.4 (3.9–10.7)		32	4.0 (3.0–7.0)		33
Pupil reaction on the field of the accident	Normal	22.7 (12.8–47.7)	0.74	20	4.7 (2.5–5.6)	0.15	20	8.0 (4.0–12.5)	0.07	24
	Abnormal	27.2 (16.1–47.1)		19	5.2 (2.7–7.3)		19	4.0 (3.0–8.0)		21
GCS14	9-15	21.6 (13.3–38.9)	0.54	23	5.3 (3.8–8.9)	0.16	25	7.5 (3.0–11.0)	0.31	26
	3-8	29.5 (16.1–52.7)		20	8.3 (5.5–11.3)		21	6.0 (3.0–8.0)		23
GOSE at 3 months	5-8	21.2 (15.3–61.1)	0.72	4	4.7 (2.9–7.0)	0.34	4	7.0 (3.3–10.5)	0.94	4
	1-4	28.9 (16.1–52.7)		27	7.2 (3.3–10.1)		30	7.0 (3.0–10.0)		33
Mortality at 3 months	Alive	21.6 (13.3–48.9)	0.40	31	5.7 (3.5–9.6)	0.11	33	7.0 (3.0–10.0)	0.78	34
	Dead	30.9 (19.6–44.3)		12	8.3 (6.0–12.3)		13	7.0 (3.0–8.5)		15
LOS ≥ 10 days for survivors in ICU	No	20.6 (9.8–31.0)	0.14	17	4.0 (3.2–7.6)	0.06	18	9.0 (5.5–11.5)	0.02	19
	Yes	27.2 (16.5–55.2)		17	9.1 (3.7–10.7)		16	5.0 (3.0–8.0)		18
LOS ≥ 10 days or death in ICU	No	20.6 (9.8–31.0)	0.06	17	4.0 (3.2–7.6)	0.007	18	9.0 (5.5–11.5)	0.03	19
	Yes	30.9 (18.6–53.8)		26	8.9 (5.4–11.3)		28	4.0 (3.0–8.0)		30
LOS ≥ 17 days for survivors in acute hospital	No	15.5 (6.9–26.7)	0.04	16	3.9 (3.2–6.4)	0.04	17	9.0 (7.0–12.0)	0.01	18
	Yes	27.2 (18.6–53.8)		17	8.8 (4.0–10.1)		17	3.0 (3.0–8.0)		18

### Relationship between MMP-9, c-Fn, f GCS and early clinical and radiological parameters

Significantly lower fGCS and a trend toward higher maximal early MMP-9 concentration or late c-Fn concentration were observed in patients with edema, with SAH or with abnormal pupil reactions on the field of accident as compared to patients without edema, without SAH or with normal pupil reactions (Table 3).

Maximal early MMP-9 concentration, late c-Fn concentration and fGCS were similar in patients with a midline shift ≥ 5 mm, with IVH or with a GOSE at 3 months ≤ 4 as compared respectively to patients with a midline shift < 5 mm, without IVH or with a GOSE at 3 months > 4 (Table 3).

### Relationship between MMP-9, c-Fn, fGCS and secondary outcomes

#### ICU LOS of survivors

Statistically lower fGCS (*p* = 0.02, *n* = 37) as well as a trend toward higher maximal early MMP-9 concentration (+32%, *p* = 0.14, *n* = 34) or late c-Fn concentration (+127%, *p* = 0.06, *n* = 34) were observed in patients staying for 10 days or more in the ICU as compared to patients who were alive and who left the ICU before 10 days (Table 3).

#### Death before 3 months

A trend toward higher maximal late c-Fn concentration was observed in patient who died before 3 months (+46%, *p* = 0.11, *n* = 46) as compared to patients who survived. Maximal early MMP-9 concentration and fGCS were similar in patients who died or survived (Table 3).

#### Consciousness at 14 days

A trend toward higher maximal late c-Fn concentration was also observed in patients with a GCS at 14 days ≤ 8 (+57%, *p* = 0.16, *n* = 46) as compared to patients with a GCS at 14 days > 8. Maximal early MMP-9 concentration and fGCS were similar in patients with a GCS at 14 days ≤ 8 or a GCS at14 days > 8 (Table 3).

#### GOSE at 3 months

No relationship was observed between MMP-9, c-Fn concentrations or fGCS and the Glasgow Outcome Scale Extended at 3 months (Table 3).

### Relationship between MMP-9, c-Fn, fGCS and the composite endpoint

Statistically lower fGCS (*p* = 0.03, *n* = 49) and higher maximal late c-Fn concentration (+123%, *p* = 0.007, *n* = 46) as well as a strong trend toward higher maximal early MMP-9 concentration (+50%, *p* = 0.06, *n* = 43) were observed in patients dying or staying for 10 days or more in the ICU as compared to patients who were alive and who left the ICU before 10 days (Table 3).

**Figure 3:**
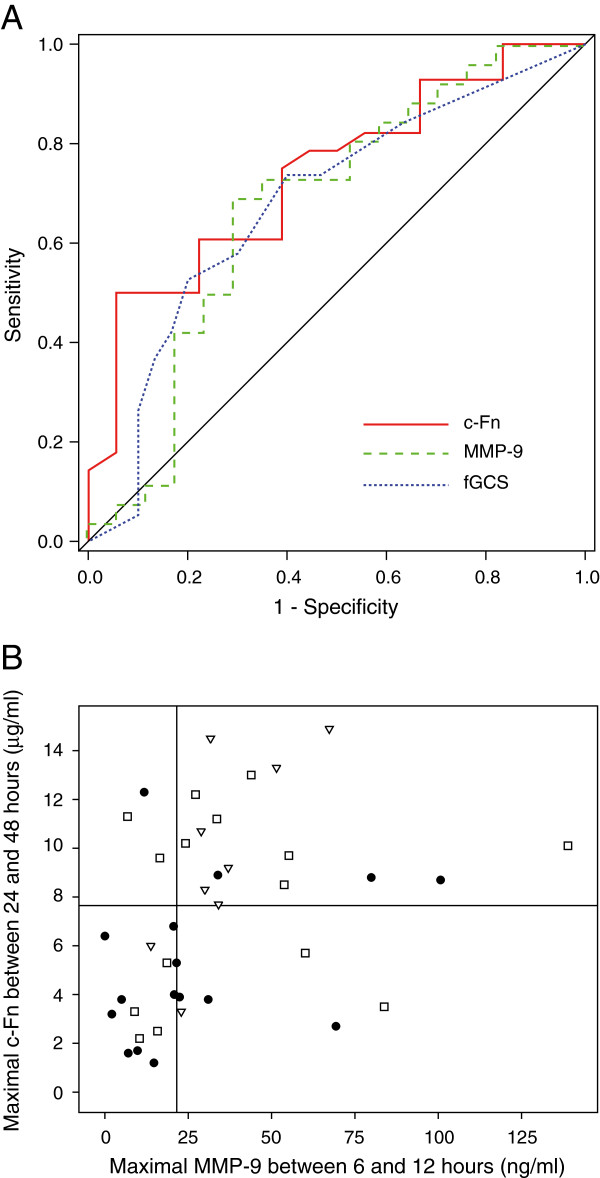
**ROC curves and relationship between MMP-9 and c-Fn concentrations and ICU LOS or death. A**: Roc curve analysis of maximal MMP-9 concentration between 6 and 12 hours, maximal c-Fn concentration] between 24 and 48 hours and fGCS for the prediction of ICU LOS ≥ 10 days or death in the ICU. Plain line: maximal c-Fn concentration between 24 and 48 hours; dash line: maximal MMP-9 concentration between 6 and 12 hours; dot line: fGCS; diagonal line: reference line. **B**: Relationship between MMP-9 and c-Fn concentrations and ICU LOS or death. Maximal MMP-9 between 6 and 12 hours was plotted against maximal c-Fn concentrations between 24 and 48 hours. Horizontal line: 7.65 μg/ml threshold; vertical line: 21.55 ng/ml threshold; circles: survivors leaving the ICU before 10 days; squares: survivors leaving the ICU at 10 days or later; triangles: patients who died in the ICU.

ROC curves for discriminating patients who died or stayed longer than 9 days in the ICU (Figure 3A) showed a slightly higher area under the curve (AUC) for maximal late c-Fn concentration (AUC = 0.737) than fGCS (AUC = 0.682) or maximal early MMP-9 concentration (AUC = 0.674). However, the ROC curves were not statistically different (bootstap test).

There was a 1.8 greater probability of dying or of staying longer than 9 days in the ICU for patients with a maximal late c-Fn concentration ≥ 7.7 μg/ml (OR, 5.4; 95% CI, 1.4-20.8; *p* = 0.01; sensitivity/specificity: 60.7%/77.8%, Figure 3B), and a 2.0 greater probability for patients with a maximal early MMP-9 ≥ 21.6 ng/ml (OR, 5.0; 95% CI, 1.3-18.6; p = 0.02; sensitivity/specificity: 73.1%/64.7%, Figure 3B). The risk of dying or of staying longer than 9 days in the ICU was 1.9 time higher for patients with a fGCS ≤ 8 as compared to patients with a fGCS > 8 (OR, 4.4; 95% CI, 1.2-15.8; p = 0.02; sensitivity/specificity: 80.0%/52.6%).

The association of c-Fn or MMP-9 with fGCS for predicting mortality or a LOS longer than 9 days in the ICU decreased dramatically the sensitivity of the tests (maximal late c-Fn concentration ≥ 7.7 μg/ml and fGCS ≤ 8: OR, 5.0; 95% CI, 1.2-20.8; *p* = 0.03; sensitivity/specificity: 48.3%/84.2%; maximal early MMP-9 ≥ 21.6 ng/ml and fGCS ≤ 8: OR, 4.0; 95% CI, 1.1-15.4; *p* = 0.04; sensitivity/specificity: 53.6%/77.8%).

## Discussion

Our data confirm the initial raise of plasma MMP-9 concentration after TBI in humans as reported by other investigators based on animal models. To our knowledge, this is the first report of a raise of plasma c-Fn concentration in TBI patients and first observation of a temporal relationship between plasma MMP-9 concentration and plasma c-Fn concentration. Furthermore it is the first time that a correlation analysis is performed between MMP-9, c-Fn, fGCS and TBI patient outcome. MMP-9, c-Fn and fGCS have similar predictive values for the composite patient-relevant endpoint ICU LOS and death. In contrast, associating the blood markers with fGCS for predicting ICU LOS and death rendered the tests ineffective with sensitivities close to 0.5.

In a rat TBI model, the local brain MMP-9 level was highest at 24 h after trauma with a second peak at 72 hours [24]. In a second rat TBI model, the local brain ProMMP-9 (the latent form of MMP-9) was highest at 6 h and lower levels were observed at 24 h [25]. In a third rat TBI model, the local brain MMP-9 activity was highest at 24 hours [26]. In our patients, the maximal plasma MMP-9 levels were at 6 and 12 hours. This difference may be species-related or due to different trauma mechanisms.

Early MMP-9 increase may cause BBB degradation leading to later c-Fn release, therefore, a relationship between maximal early MMP-9 concentration and subsequent maximal c-Fn concentration is not surprising. This temporal profile of MMP-9 and c-Fn concentrations supports a role for MMP-9 in fibril degradation after TBI. Both MMP-9 and c-Fn concentrations tend to be higher in patients with brain oedema. The cause of MMP-9 increase after TBI has not been investigated in our study but could be related to inflammation and neutrophil activation [41,42] after injury. Activated MMP-9 degrades c-Fn, which is released into circulation as a marker of clinical severity as suggested by the trend toward higher maximal c-Fn concentration between 24 and 48 hours in patients with a GCS at 14 days ≤ 8 and in patients that died before 3 months.

The risk of longer ICU LOS (≥ 10 days) for survivors or death in the ICU was statistically increased in patients with a maximal MMP-9 concentration equal of higher than 21.6 ng/ml between 6 and 12 hours after TBI or with a maximal c-Fn equal or higher than 7.7 μg/ml between 24 and 48 hours. A statistically significant relationship between this composite endpoint and fGCS equal or lower than 8 was also observed. Patients with severe TBI have two pattern of LOS in hospital: Non-survivors with a short median hospital LOS of 3 days and survivors with a long median hospital stay of 24 days [43]. ICU LOS is directly related to the clinical condition of the patients. MMP-9 and c-Fn concentration measurements might be more objective and reliable tools than fGCS for the prediction of the ICU LOS or death because the considerable inaccuracy in GCS scoring in daily practice [10].

Early prognostic factors of hospital LOS after severe TBI were rarely investigated but could have clinical implications. Apart the short LOS of patients with fatal injuries [43], post-traumatic sepsis and pneumonia were considered as risk factors for prolonged hospital LOS [44]. Early predictors such as MMP-9 and c-Fn may be useful for enhanced in-hospital health care resources planning and improved patient and family information. Furthermore, ICU LOS is a relevant predictor for rehabilitation LOS after traumatic brain injury underlying the desirability of improved hospital LOS prediction [45].

This study has several limitations. First, the significant relationship between early brain-derived biomarkers sampled in blood and the composite endpoint will not mean causality between the degree of TBI and patients’ outcome. Second, inclusion criteria was not defined with the physiological instrument GCS, but with the anatomical instrument HAIS. This cohort of 49 patients had, therefore, included patients with different states of consciousness with a median fGCS 7 and median GCS 3 in the emergency department. The inclusion criteria of this study was based on the AIS trauma system to avoid biases related to clinical interventions and inter-rater variability [10]. Third, our results have to be considered as a proof-of-concept. In contrast to animal studies, patients with TBI have a heterogeneous brain injury pattern and different sizes of injuries with different quantity of release of biomarkers making relationship with clinical outcomes less evident. The absence of relationship for the component end points alone and the other secondary outcomes in our cohort of 49 patients will not exclude any association in a more homogeneous or larger population. Probably, a very high number of patients would be needed to test association for a single patient-relevant outcome. Composite endpoint provides evidence for a hypothesis with fewer patients, but may be criticised. However, there is a biological basis for our results: Brain edema, a major cause of secondary injury after TBI [46,47], is partly due to MMP-9 activation and degradation of components of the basal lamina such as c-Fn, leading to blood–brain barrier disruption [20] and to severe TBI with prolonged ICU stay and death.

The clinical relevance of our results is actually limited despite an association between the biomarkers MMP-9 and c-Fn and a clinical composite endpoint. However, this clinical study has confirmed some postulated mechanisms in the early phase of TBI the first time in humans. Therefore, there is a potential that these early plasmatic biomarkers may predict patients’ outcome. Validation of our results with other more homogenous and larger TBI populations is needed before clinical implementation may be considered.

## Conclusion

In conclusion, the plasma biomarkers MMP-9 and c-Fn can be measured in the early phase after TBI and are predictive for the composite endpoint of ICU LOS in survivors and death but not for consciousness at 14 days nor outcome at 3 months.

## Competing interests

The authors declare that they have no competing interests.

## Authors’ contributions

JCC and BW contributed to the conception of the study, to the acquisition and analysis of the data, and drafted the manuscript. MR participated in the acquisition of the data. NT and XR helped in statistical analysis, in interpretation of the results and in the editing of the manuscript. JCS, KS and YG helped in interpretation of the results and in the editing of the manuscript. All authors have read and approved the final version of this manuscript.
